# How do invasive predators and their native prey respond to prescribed fire?

**DOI:** 10.1002/ece3.11450

**Published:** 2024-05-22

**Authors:** Darcy J. Watchorn, Tim S. Doherty, Barbara A. Wilson, Mark J. Garkaklis, Don A. Driscoll

**Affiliations:** ^1^ School of Life and Environmental Sciences (Burwood Campus) Deakin University Geelong Victoria Australia; ^2^ School of Life and Environmental Sciences The University of Sydney Sydney New South Wales Australia; ^3^ Biodiversity and Conservation Science Department of Biodiversity, Conservation and Attractions Woodvale Western Australia Australia; ^4^ State of the Environment Pty Ltd Aireys Inlet Victoria Australia

**Keywords:** activity, camera trap, conservation, disturbance, feral cat (*Felis catus*), macropod, mammal, predation, red fox (*Vulpes vulpes*), small mammals

## Abstract

Fire shapes animal communities by altering resource availability and species interactions, including between predators and prey. In Australia, there is particular concern that two highly damaging invasive predators, the feral cat (*Felis catus*) and European red fox (*Vulpes vulpes*), increase their activity in recently burnt areas and exert greater predation pressure on the native prey due to their increased exposure. We tested how prescribed fire occurrence and extent, along with fire history, vegetation, topography, and distance to anthropogenic features (towns and farms), affected the activity (detection frequency) of cats, foxes, and the native mammal community in south‐eastern Australia. We used camera traps to quantify mammal activity before and after a prescribed burn and statistically tested how the fire interacted with these habitat variables to affect mammal activity. We found little evidence that the prescribed fire influenced the activity of cats and foxes and no evidence of an effect on kangaroo or small mammal (<800 g) activity. Medium‐sized mammals (800–2000 g) were negatively associated with prescribed fire extent, suggesting that prescribed fire has a negative impact on these species in the short term. The lack of a clear activity increase from cats and foxes is likely a positive outcome from a fire management perspective. However, we highlight that their response is likely dependent upon factors like fire size, severity, and prey availability. Future experiments should incorporate GPS‐trackers to record fine‐scale movements of cats and foxes in temperate ecosystems immediately before and after prescribed fire to best inform management within protected areas.

## Graphical abstract

There is growing concern that the hunting success of invasive mammalian predators increases shortly after prescribed burns. We investigated how invasive mammals and their native prey respond to a prescribed fire. In contrast to our predictions, we found little evidence that the prescribed fire influenced the activity of cats and foxes. However, medium‐sized mammals (800–2000 g) were negatively associated with prescribed fire extent, suggesting that these species may be vulnerable to prescribed fire in the short term.

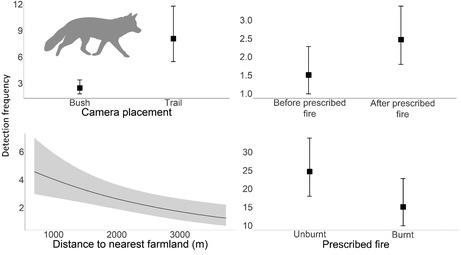

## 1 INTRODUCTION

Terrestrial mammal species are experiencing rapid and significant declines across the globe (Brodie et al., 2021; Di Marco et al., 2014; Woinarski et al., 2015). In many cases, protected areas are providing the last population strongholds (Geldmann et al., 2013; Pacifici et al., 2020), however, these areas are often vulnerable to pervasive ecological threats, particularly fire and invasive species (Lawes et al., 2015; McCain, 2019; Rija et al., 2020; Tedeschi et al., 2022). Fire is a fundamental ecological process in many ecosystems (McLauchlan et al., 2020; Pereira et al., 2012). It alters vegetation structure, floristics, and soil nutrients, and in doing so affects resource availability for animals across multiple spatial and temporal scales. Yet fire regimes, defined as the pattern, frequency, and intensity of fires, have undergone relatively rapid changes in many ecosystems (Bowman et al., 2011). These alterations are due, at least in part, to shifts in prescribed burning practices (Bird et al., 2020; Fernandes & Botelho, 2003; Mariani et al., 2022) and an increased frequency and/or severity of wildfire, driven by the proliferation of exotic grasses (Fusco et al., 2019), and, increasingly, human‐induced climate change (Canadell et al., 2021; Jolly et al., 2015; Van Oldenborgh et al., 2020). Altered fire regimes can profoundly influence ecosystems, particularly through their effects on the occurrence and interactions of invasive and native species (Doherty et al., 2024; Nunes et al., 2020; Perry et al., 2015; Stritar et al., 2010). Therefore, understanding how both wildfire and prescribed fire affect the ecology of an area is crucial for managing and conserving biodiversity.

Invasive mammalian predators are amongst the most damaging invasive species groups (Doherty et al., 2016; Hilton & Cuthbert, 2010). Species such as the feral cat (*Felis catus*), stoat (*Mustela erminea*), small Indian mongoose (*Urva auropunctata*), and European red fox (*Vulpes vulpes*) have become major threats to biodiversity outside their native ranges (Doherty et al., 2016; GISD, 2023). These species are highly adaptable and opportunistic and can thrive across a range of landscapes, including within large remnant forests, along edges of fragmented forests, in agricultural and urban environments, and many other ecosystems (Alexandre et al., 2020; Fisher & Wilkinson, 2005; Louppe et al., 2020; Nichols et al., 2019). The occurrence and relative abundance (i.e. activity) of these predators can increase in response to prey abundance (Scroggie et al., 2018), recent burning (Birtsas et al., 2012; Doherty et al., 2023; Nalliah et al., 2022), and anthropogenic features (such as tracks and farms) that provide efficient movement and foraging opportunities in structurally complex environments like forests (Colón & Kamil, 2020; May & Norton, 1996; Schwemmer et al., 2021). Ecosystem‐specific knowledge of how invasive predators respond to these factors can assist land managers in developing more targeted strategies to effectively mitigate their impacts (e.g., McGregor et al., 2020).

The response of mammals to fire is commonly driven by how fire alters their food and shelter resources (Griffiths & Brook, 2014; Lees et al., 2022; Morris et al., 2011; Puig‐Gironès, 2023; Torre et al., 2023), rather than direct mortality (Hale et al., 2022; Shaw et al., 2021). For instance, some small mammals require habitat characterised by high vegetation complexity and productivity (Sukma et al., 2019) due to food availability and shelter from predators (Hanser et al., 2011; McCain et al., 2018; Swan et al., 2020). Consequently, these species may suffer reduced fitness shortly after fire. For instance, the Trowbridge's shrew (*Sorex trowbridgii*) in North America, declined after fire in response to a loss of food or an increased predation risk (Culhane et al., 2022; Greenberg et al., 2007). Conversely, the abundance of larger herbivores, such as the red‐flanked duiker (*Cephalophus rufilatus*) and zebra (*Equus quagga*) in Africa, can increase in recently burnt areas, as the regenerating vegetation is typically more palatable and the risk of predation by ambush predators, such as lions (*Panthera leo*), is lower (Funston et al., 2001; Klop et al., 2007; Reid et al., 2023). Yet despite an increase in food availability, some herbivores, such as the white‐tailed deer (*Odocoileus virginianus*) in North America, will avoid recently burnt areas, likely due to an increased risk of predation by the cursorial coyote (*Canis latrans*), which benefit from the increased exposure of their prey (Cherry et al., 2017).

Fire‐driven changes in predator–prey interactions are especially likely to be detrimental to native fauna when they benefit invasive mammalian predators (Doherty et al., 2022; Geary et al., 2020). The feral cat (hereafter ‘cat’) now has a near worldwide distribution and is a significant threat to many native species globally, whereas the European red fox (hereafter ‘fox’) represents a particular threat in Australia (Loss et al., 2013; Stobo‐Wilson et al., 2022). Indeed, both are amongst the world's top 100 most damaging invasive species (GISD, 2023). Whilst their severity and impact on vegetation structure are often different, both prescribed fire and wildfire can increase the activity of these two predators by removing vegetation, increasing the visibility and availability of prey (Birtsas et al., 2012; Hradsky, 2020; Leahy et al., 2016; McGregor et al., 2015; Miritis et al., 2023; Puig‐Gironès & Pons, 2020). Both species hunt a range of small vertebrates (Doherty, Davis, et al., 2015; Fleming et al., 2021; Woolley et al., 2019), whereas foxes are also capable of predating larger mammalian herbivores, such as kangaroos and deer—particularly in open environments (Banks, 2001; Banks & Dinnel, 2000; Panzacchi et al., 2009). Although foxes are typically cursorial predators and cats typically ambush, both species can be opportunistic and adaptive hunters (Moseby & McGregor, 2022; Tobajas & Díaz‐Ruiz, 2022) and may experience improved hunting efficiency in burnt compared to unburnt areas (i.e., habitat‐specific predator lethality), likely due to increased prey visibility and availability of edge habitat (Doherty et al., 2022; McGregor et al., 2015).

Climate change is likely to continue to intensify the effects of fire in many forested areas globally, by potentially leading to more frequent, large, and/or severe wildfires (Canadell et al., 2021; Jones et al., 2022; Wasserman & Mueller, 2023). This trend is likely to be paralleled by a rise in the use of prescribed fires in an effort to mitigate the escalating threat to human life and infrastructure posed by these intensified wildfire events (Kolden, 2019; Varner et al., 2021). Such changes may also facilitate range expansions for both cats and foxes (Aguilar et al., 2015; Elmhagen et al., 2017) and consequently increase the likelihood of negative impacts on native fauna. As such, well‐informed management strategies that account for the effects of fire, vegetation, and anthropogenic features on the activity of cats, foxes, and native mammals, are needed so the effectiveness of protected areas for conservation can be optimised.

Australia serves as one of the most prominent global examples where the potential for the interaction between fire and invasive predators carries a great risk for native mammals (Doherty et al., 2023). Since European colonisation, many overlapping threats have contributed towards the decline of Australia's mammal community (Ashman et al., 2021; Legge et al., 2023; Woinarski, Braby, et al., 2019). The cat and fox have played a particularly damaging role, contributing to the extinction of >25 mammal species and killing an estimated 556 million native mammals each year (Kearney et al., 2019; Stobo‐Wilson et al., 2022; Woinarski, Legge, & Dickman, 2019). Not all Australian studies have found that cat and fox activity increases after fire (e.g., Bird et al., 2018; Hradsky, Penman, et al., 2017; Lothian et al., 2022), and a quantitative review of this research found that—should this phenomenon be observed—it was most likely to occur shortly after fire (e.g., weeks to months; Doherty et al., 2023). Studies from Greece and Catalonia, where the red fox is native, have also found similar relationships (Birtsas et al., 2012; Puig‐Gironès & Pons, 2020). This indicates that there may be a critical period immediately post‐fire when prey is most vulnerable to an elevated risk of predation.

The occurrence of wildfire is typically unpredictable, and safety and logistical issues can prohibit researcher access for several weeks or even months. Thus, there is an inherent difficulty in undertaking surveys shortly before and after wildfire during this potentially critical time period. Whilst prescribed fires often differ from wildfire in severity, scale, and season of occurrence, they offer researchers opportunities to conduct targeted field experiments and access fire grounds early—potentially within days. To this end, we examined the effect of a prescribed fire on the activity (defined as detection frequency) of invasive cats, foxes, and the native mammal community within a conservation reserve in south‐eastern Australia. Specifically, we investigated how prescribed fire interacts with environmental variables—such as vegetation type, topography, fire history, and proximity to anthropogenic features like towns and farms—to understand their impact on invasive predators and their prey, and identify effective conservation opportunities for land managers. We used camera traps to quantify mammal activity across 30 sites, both before and immediately after a prescribed fire at burnt and unburnt sites. We fitted generalised linear mixed models to test the following predictions:

1. The invasive cat and fox will be more active at sites burnt by the prescribed fire (Hradsky, Mildwaters, et al., 2017; McGregor, Legge, et al., 2016), near anthropogenic features (Hradsky, Robley, et al., 2017; Schwemmer et al., 2021), and where mammalian prey activity is higher (Geary et al., 2022).
2. Despite the increased availability of regenerating vegetation preferred for grazing (Klop et al., 2007; Reid et al., 2023), the native macropod community (kangaroos and wallabies) will show decreased activity in areas burnt by the prescribed fire, due to the increased risk of predation from foxes (Cherry et al., 2017).
3. The activity of the native small and medium mammal (<2 kg) community will be negatively influenced by the prescribed fire due to fewer shelter resources and an increased predation risk. They will also be positively influenced in riparian and highly productive areas (Lawes et al., 2015; Mariani et al., 2022; Swan et al., 2015).

## 2 METHODS

### 2.1 Study area

**FIGURE 1:**
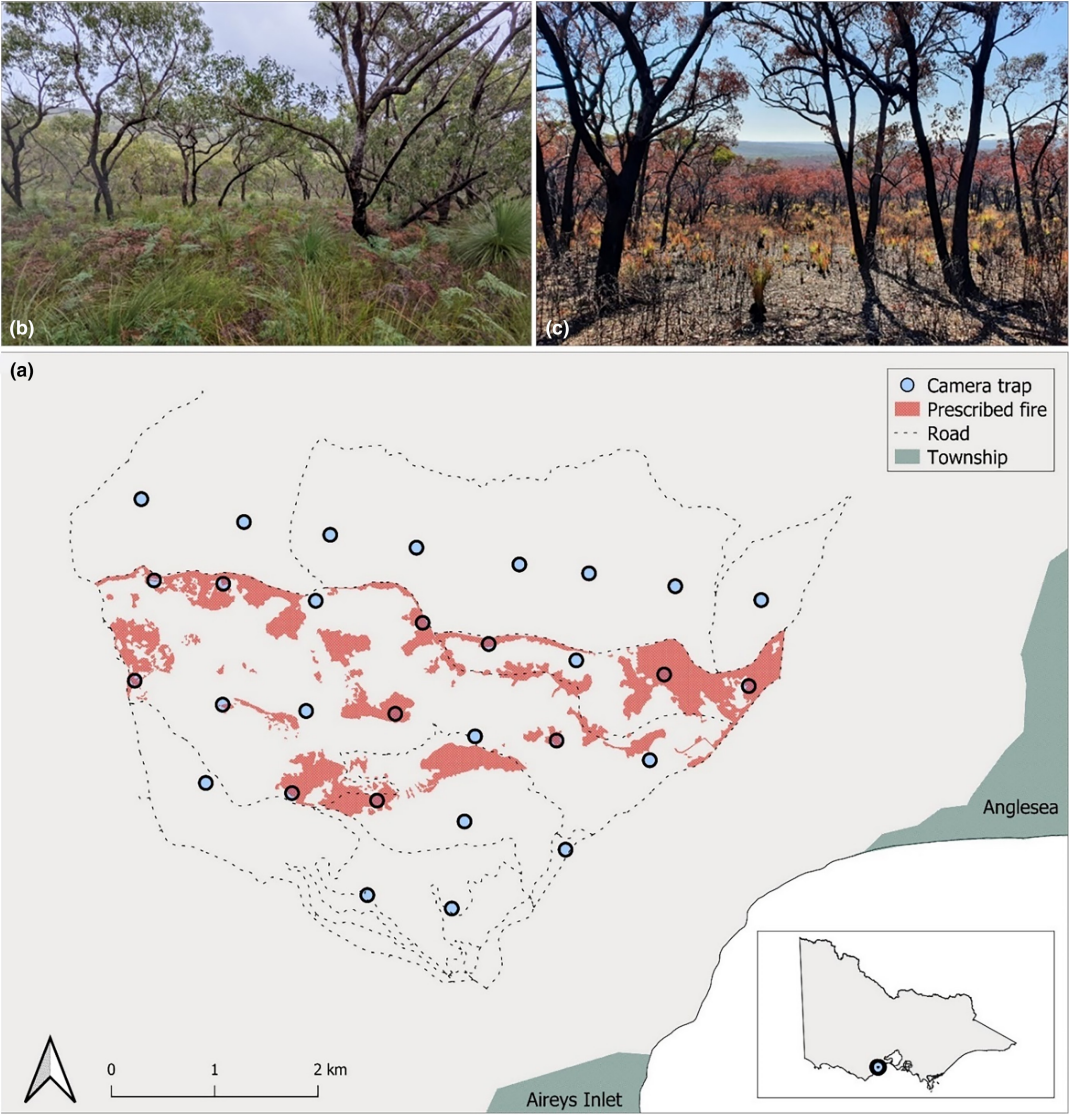
(a) A map of the study area in the north‐eastern Otway Ranges, showing the placement of the camera trap (*N* = 30) grid and the extent of the prescribed burn across the site and within the 100 m radius of each of the affected camera traps (*N* = 12). The prescribed burn occurred in May 2019. (b) a photograph of one of the heathy woodland sites from the study area before the prescribed fire. (c) a photograph of the same heathy woodland site approximately 4 weeks after the prescribed fire.

Our study was conducted in the northeast region of the Great Otway National Park, a high‐value conservation reserve which forms part of the Otway Ranges in Victoria, Australia (38°24′ S, 144°1′ E) (Parks Victoria, 2020). The 2471‐hectare study area comprises eucalypt woodlands, heathlands, and wet shrublands (Figure 1). This part of the Otway Ranges has a mean maximum temperature of 18.4°C and an average annual rainfall of 627 mm (BoM, 2021). The dominant overstory vegetation includes messmate (*Eucalyptus obliqua*) and brown stringybark (*E. baxteri*), with common mid‐storey and ground cover species including the myrtle wattle (*Acacia myrtifolia*), prickly tea tree (*Leptospermum continentale*), and austral grass tree (*Xanthorrhoea australis*). The Traditional Owners of this land are the Wadawurrung People.

The Great Otway National Park supports a diverse terrestrial mammal community which has suffered considerable decline in recent decades, including the near‐threatened long‐nosed potoroo (*Potorous tridactylus*), the vulnerable swamp antechinus (*Antechinus minimus maritimus*) and white‐footed dunnart (Sminthopsis leucopus), and the endangered southern brown bandicoot (*Isoodon obesulus*) (Wayne et al., 2017; Wilson & Aberton, 2006; Wilson et al., 2001, 2017; Wilson & Garkaklis, 2020). Foxes and cats predate all these species (Fleming et al., 2021; Stobo‐Wilson et al., 2021, 2022; Wolrige, 2000; Woolley et al., 2019) and are the largest terrestrial predators in the region. Fox control, using baits containing 1080 poison (sodium fluoroacetate), is regularly undertaken by land managers, although the impact on fox occurrence is likely modest (Robley, Fanson, and PV, 2019). There is no broad‐scale management of cats.

The regions' fire regime is characterised by regular prescribed burns and infrequent large, severe wildfires. Major wildfires burnt the study area in 1939 (Black Friday; 240,000 ha) and 1983 (Ash Wednesday; 40,000 ha). Prescribed fire is regularly applied in the drier forest types (Table 1) aimed at limiting the risk of wildfire to nearby towns (Figure 1), which experience significant seasonal increases in population during the summer months (Gazzard et al., 2020; ODBPC, 2021). The vegetation types in this region have a similar fire return interval (mean: 35 years, [range: 31–41 years; Table 1]) and much of the study area experienced 1–2 prescribed burns between 1988 and 2018 (DEECA, 2022).

**TABLE 1:** Descriptions of the predictor variables included in our generalised linear mixed models of mammal activity in the eastern Otway Ranges, Victoria.

Variable (*abbr*.)	Description
Detection	
Camera placement (CP)	Cameras were placed on trails (i.e. well‐established gamepads or discontinued walking trails/vehicle tracks, *N* = 7), or off trails in bushland (*N* = 23)
Age of lure (AoL)	Mean (x̄) number of days (per survey period) since lures were replaced. Lure freshness can influence the detectability of some species, particularly predators (Mills et al., 2019). Lures were replaced at the start of surveys 1, 2, and 3. Due to site access issues, lures were replaced x̄ 26 days into survey 4 (when the lures were x̄ 90 days old). The lures were not replaced for the remainder of the experiment, meaning that lures were x̄ 38 days old at the start of survey 5
Fire history	
Years since previous fire (YSPF)	Number of years since the site was last burnt by prescribed or wildfire (i.e., prior to the 2019 prescribed fire) (DEECA, 2022). This variable was chosen as it affects the vegetation successional stage and thus structural complexity and resource availability for mammals; although we note that fire severity (not included as a predictor variable due to the resolution and patchiness of available data for some areas DEECA [2022]) will also influence these factors
Number of fires (NoF)	Number of fires (incl. prescribed and wildfire) that have affected each site within the last 100 years, prior to the 2019 prescribed fire (DEECA, 2021a). An inappropriately high frequency of fires (relative to historic baseline fire regimes) can lead to simplified forest structure and reduced habitat complexity and availability for native mammals. For the four vegetation types included in this study, the mean fire interval and number of fires (DEECA, 2022) in this timeframe were: Dry Forest: 31‐year interval, 2.5 fires (1.8 wildfires, 0.7 prescribed burns). Heathy Woodland: 32‐year interval, 3.1 fires (1.7 wildfires, 1.4 prescribed burns). Lowland Forest: 37‐year interval, 2.9 fires (1.8 wildfires, 1.1 prescribed burns) Swampy Riparian Woodland: 41‐year interval, 2.3 fires (1.6 wildfires, 0.7 prescribed burns).
2019 prescribed fire	
Before‐after (BA)	Before cf. after prescribed fire. Two surveys pre‐fire, three surveys post‐fire
Treatment (CI)	Burnt cf. unburnt. 12/30 sites were burnt by the prescribed fire
Fire extent (FireExt)	The percentage of burnt area at each site within a 100 m radius of the camera (approx. 3‐ha). All sites had a value of 0% for the first two (pre‐fire) surveys
Vegetation	
Vegetation type (VT)	One of four categories: lowland forest, heathy woodland, swampy riparian woodland, or dry forest. We created these categories from condensing ecologically similar ecological vegetation classes. VT reflects species' food and shelter requirements (Lees et al., 2022; Norton et al., 2015; Swan et al., 2015)
Normalised difference vegetation index (NDVI)	NDVI is a remotely sensed measure of vegetation productivity that is positively related to photosynthetic activity, green leaf biomass, fraction of green vegetation cover, and primary productivity (Myneni et al., 1995; Tucker et al., 1986). NDVI ranges from −1 to +1, and is a widely used metric for quantifying the health and density of vegetation, which can be a useful predictor of mammal occurrence (Campbell‐Jones et al., 2022). We calculated the mean NDVI within 50 m of each site, selected from the closest available Landsat 8 satellite imagery. For the year 2019, we had access to only one NDVI layer that met our criteria of no cloud cover and fell within a relevant timeframe for our surveys. This image was then used to approximate the vegetation conditions across the duration of our study period
Topography	
Topographic position index (TPI)	A measure of the relative height of each camera site (i.e., topographic ruggedness) compared to the terrain within 100 m, derived from a DEM at 10 m resolution. For example, a TPI value of −35 indicates that the location is 35 m lower in height than the average height in the surrounding 100 m. TPI can represent site productivity, which influences occurrence of some mammal species (Moore et al., 2019). TPI was preferred over elevation as a topographic variable, due to the modest elevational gradient in the eastern Otways
Anthropogenic features	
Distance nearest township (DNT)	The Euclidean distance (m) from each site to the nearest mapped township (DEECA, 2021b) and farmland (DEECA, 2021c)
Distance nearest farm (DNF)	
Prey activity	
Small mammal (SM)	Detections of SM (<2 kg) and LM (>2 kg) per site, standardised for survey effort. Mammalian prey availability can influence feral cat and red fox activity patterns (McGregor et al., 2014)
Large mammal (LM)	

*Note*: The spatial data from the 2019 prescribed fire was sourced from DEECA (2022).

### 2.2 Study design

Forest Fire Management Victoria conducted a prescribed burn within our study area in mid‐May 2019. The fire affected 292 ha, of which 246 ha was forest and 46 ha was heathland (DEECA, 2022). The majority of the fire (66%) burned at low‐medium severity (DEECA, 2022), although severity was not quantified for heathland areas. We conducted five repeated mammal surveys using camera traps with infrared flash (Reconyx HF2X) across 30 sites (Figure 1) over a 12‐month period. Surveys were conducted at 6‐ and 2 months pre‐fire and 2 weeks, 3 months, and 6 months post‐fire, with each survey period being approximately 2 months in length (Appendix S1: Table S1). Using a Geographic Information System, we determined camera trap locations by positioning a grid of 30 camera survey sites over the study area, where each grid point/camera site was separated by 900 m (ESRI, 2014). During deployment, some camera traps were moved up to 150 m from grid points to account for access issues or to target nearby game trails or old vehicle/walking tracks, as feral cat and fox detectability is generally higher on trails compared to off trails (Geyle et al., 2020). We did not place cameras on public roads or heavily used walking trails to reduce the risk of theft.

Of the 30 camera trap sites, 40% (12/30) were burnt during the prescribed burn (Appendix S1: Table S1; Figure 1), and the mean area burnt within a 100 m radius (i.e., Fire extent, Table 1) for these sites was 54% (range 34%–95%); highlighting the patchy burning style that is common of prescribed fires in our study region (e.g., Hradsky, Mildwaters, et al., 2017; Sitters et al., 2015). The burnt and unburnt sites were not spaced far enough apart to be considered independent for all of our study species (i.e., they were within the feasible movement range of some species). Therefore, our study design would be more appropriately described as a quasi‐BACI (before‐after, control‐impact) design, acknowledging this potential for spatial dependence between the sites.

At each site, cameras were attached to a tree at a height of approximately 40 cm facing a lure station two metres away. Each lure station was comprised of wadding soaked in tuna oil encased in a polyvinyl chloride (PVC) vent cowl which was pegged securely into the ground. Lures are commonly used when surveying for cryptic predators—especially felids and canids—to increase detection probability and analytical power, particularly when cameras are set off trails (Cove et al., 2014; Ferreras et al., 2018; Rees et al., 2019; Satterfield et al., 2017). We acknowledge, however, that lures may alter space use and local movement patterns of nearby animals, potentially causing an over‐ or underestimate of certain species' presence (Da Rocha et al., 2016; Gil‐Sánchez et al., 2021; Johnson et al., 2021). We incorporated lure age into our analytical framework due to its potential to influence detectability (Stobo‐Wilson, Brandle, et al., 2020; see Section 2.3), and we suggest that these factors be considered when interpreting the results. We also acknowledge that the distance between the camera and the lure was greater than ideal for detecting and identifying small mammals (Burns et al., 2017) due to the size range of the target species in this study. To mitigate any impact this may have on small mammal detectability, the vegetation in each camera's line of sight was cleared to ensure animals were clearly visible, as well as to prevent false triggers. We also set cameras to trigger at medium‐high sensitivity to improve trigger likelihood, with no delay between trigger events. Only 6% of small mammal detections could not be identified to species level (Appendix S1: Table S2). Cameras recorded three images per trigger. We did not quantitatively measure vegetation structure at each site; however, our field observations indicated a slow recovery from the fire across all vegetation types, suggesting a relatively consistent habitat structure throughout each survey period.

### 2.3 Statistical analyses

Images were processed using CPW Photo Warehouses (Ivan & Newkirk, 2016). Animals were identified to species level where possible, otherwise, they were categorised according to the finest taxonomic/functional group possible (e.g., ‘unknown small mammal species’). To reduce the influence of animals spending time in front of the camera investigating the lure, a detection event was defined as images of the same species on the same camera separated by at least 60 min (Holinda et al., 2020). Species detection matrices were created using the camtrapR package (Niedballa et al., 2016) in R version 4.2.2 (R Core Team, 2022). There were many zeros (i.e., days in which a species was not detected) in the detection matrices due to long intervals between detection events. To account for this, we defined our activity response variable as the detection frequency of each species in each survey period. Detection frequency was calculated as the number of days a species was detected in a survey period relative to the number of days it was not detected. This measure provided an index of relative activity levels (rather than absolute abundance or occupancy) and accounted for each day in the survey period, and served to minimise the prevalence of zeros in the dataset—mitigating the risk of model overfitting due to infrequent detections.

To test the influence of the fire, habitat, anthropogenic, and prey variables (Table 1) on mammal activity, we fitted generalised linear mixed models (GLMMs) to each species/group with sufficient data (i.e., >150 detections; Appendix S1: Table S2). The detection frequency response variable effectively represents Bernoulli trials, thus we used the binomial distribution for all models. GLMMs were selected due to their flexibility in incorporating fixed and random effects, as well as their capacity to handle complex interactions and account for overdispersed and zero‐inflated data. This approach also allowed for a more extensive use of the detection data compared to alternative methods, such as occupancy models (Appendix S2). Notably, however, some mammals may become easier to detect in burnt areas where there is less vegetation obstructing visibility, compared to densely vegetated areas. To account for this potential to confound detection probability with activity, we initially fitted single‐species occupancy and detection models to test whether Treatment (before/after, unburnt/burnt) influenced detection probability (detailed in Appendix S2). We found no evidence that detectability increased in burnt areas (Appendix S2), thus we proceeded with using GLMMs to address our research predictions.

There were four species included in our analyses: the red fox, feral cat, swamp wallaby, and eastern grey kangaroo. To fit models and test our predictions on smaller mammals (<2000 g), we pooled detections from small mammals (<800 g) and medium‐sized mammals (800–2000 g). The species comprising these two groups (see Appendix S1: Table S2) were recorded too infrequently to fit models to individual species, and many detections were not identifiable to species level. We conducted all model fitting and verification using the glmmTMB (Brooks et al., 2017), MUMIn (Barton, 2022), and DHARMa (Hartig, 2022) packages in program R version 4.2.2 (R Core Team, 2022).

Before testing the covariates for each species/species group, we constructed models to test the effect of two possible detection covariates, namely camera placement (on or off trail) and age of lure (Table 1) on each response variable. Camera placement can influence the detectability of cats and foxes (Geyle et al., 2020), whereas the age of a lure might impact mammal activity either through reduced potency over time or behavioural alterations (Frey et al., 2017; McHugh et al., 2019). Although the five survey periods were similar in length (refer to Appendix S1: Table S1), there was inconsistency in the timing of lure replacement. Lures for surveys four and five were replaced part‐way through the survey periods, unlike those in surveys one, two, and three, which were replaced at the beginning. These detection models incorporated the main effects of both camera placement and lure age, along with random effects of Survey period and Site, allowing us to account for repeat sampling over time and any camera‐level variability. We assessed the output of these models and included camera placement and/or lure age as fixed effects in subsequent analyses if the 95% confidence intervals did not cross zero.

To test the effect of our remaining variables on mammal activity, we fitted binomial GLMMs containing three‐way interactions between Treatment (CI), Before‐After (BA) and each of the remaining non‐detection covariates (Table 1). These models included a total of 14 variables: two Fire History variables, three variables relating to the 2019 Prescribed Fire, two Vegetation variables, one Topography variable, two variables representing proximity to Anthropogenic Features, and potentially one or both of the Detection variables if they influenced the activity of the species/group (Table 1). We included both the Large mammal and Small mammal Prey Activity variables (Table 1) for the fox, and the Small mammal Prey Activity variable for the cat. We limited the maximum number of variables per model to 10 to avoid issues associated with overfitting, which meant that only one three‐way interaction could feature in any given model. We used the ‘dredge’ function from the MuMIn package for model selection. This function only allows a maximum of 31 variables in the global model, including interaction terms. Due to the complexity arising from fitting three‐way interaction between BA, CI, and most of the aforementioned variables, we fitted two unique ‘sub‐global’ models containing different sets of variables, each with <31 terms. We then used the ‘merge.model.selection’ function to combine the two model selection tables per species/group and reranked the models by AICc. The selection criteria for well‐supported models were based on a delta Akaike Information Criterion (ΔAICc) of less than 2 (Burnham & Anderson, 2004).

For the Fire extent variable, we fitted a simplified two‐way interaction with BA. This is because all unburnt sites had a Fire extent of 0%, making it redundant to include the CI variable. We did not fit an interaction between BA or CI and Vegetation Type, Years since previous fire, or Fire Frequency, as this resulted in model convergence issues. Moderately and highly correlated variables (i.e., Pearson's *r* ≥ .5) were not included in the same model. Additionally, we excluded normalised difference vegetation index (NDVI) and topographic position index (TPI; refer to Table 1 for a description of TPI and NDVI) from appearing in the same model (Pearson's *r* = .49) as high NDVI values frequently coincide with gullies and riparian areas, which are characterised by lower TPI values (e.g., Svoray & Karnieli, 2011). We included the random effects of Site and Survey Period as per the initial detection models, and we scaled and centred each of the continuous predictor variables prior to modelling. We present the coefficients from the most well‐supported models in the Results and provide the rankings, estimates, and 95% confidence intervals of each well‐supported model in Appendix S3.

## 3 RESULTS

### 3.1 Detection summary

We recorded 4476 mammal detections events, of which 132 (2.95%) were feral cats, 286 (6.39%) were red foxes, and 4058 (90.66%) were native animals comprising 17 species (Table S1). The most frequently detected native species was the swamp wallaby (*Wallabia bicolor*) (3231 detections, 72.19%), followed by the eastern grey kangaroo (*Macropus giganteus*) (290 detections, 6.48%), and bush rat (*Rattus fuscipes*) (93 detections, 2.08%; Table S1).

### 3.2 Predictors of invasive predator activity

**FIGURE 2:**
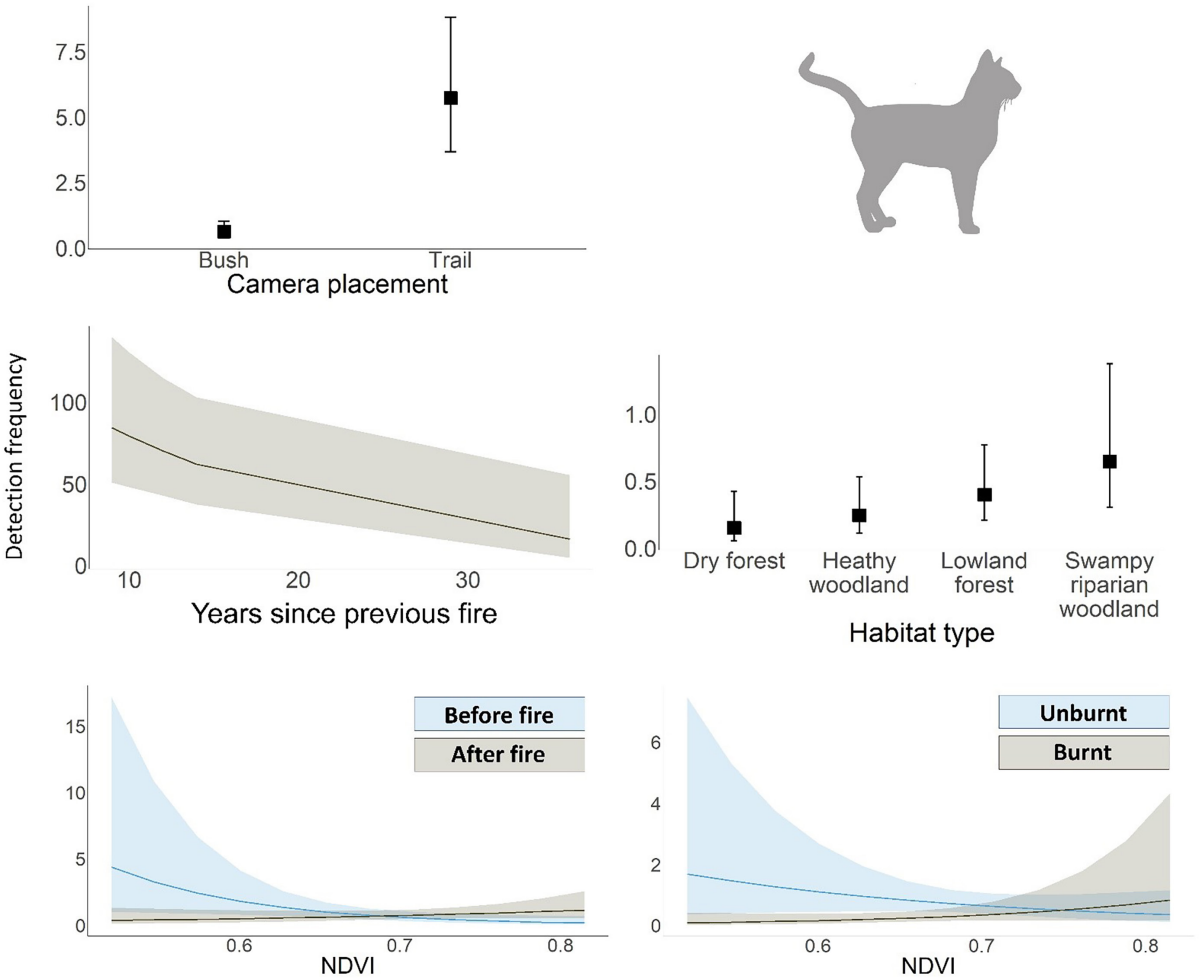
Plots of the GLMMs showing the effects influencing cat activity (detection frequency) in the two well‐supported models (ΔAIC < 2) (Appendix S3).

There were six well‐supported models for cats (Appendix S3). Cat activity was higher on trails compared to off trails (*β* [95% CI] = 1.86 [1.37, 2.34]; 6/6 models), negatively associated with Years since previous fire (−0.70 [−1.09, −0.30]; 6/6 models), and higher in swampy riparian woodland vegetation type (0.50 [0.01, 1.00], 2/6 models) (Figure 2). There were also two interactions present; cat activity was negatively associated with NDVI before the prescribed fire (−0.86 [−1.41, −0.32]; 6/6 models) and in unburnt areas (−0.71 [−1.39, −0.03]; 2/6 models) (Figure 2). There was no evidence that cat activity was influenced by prey activity.

**FIGURE 3:**
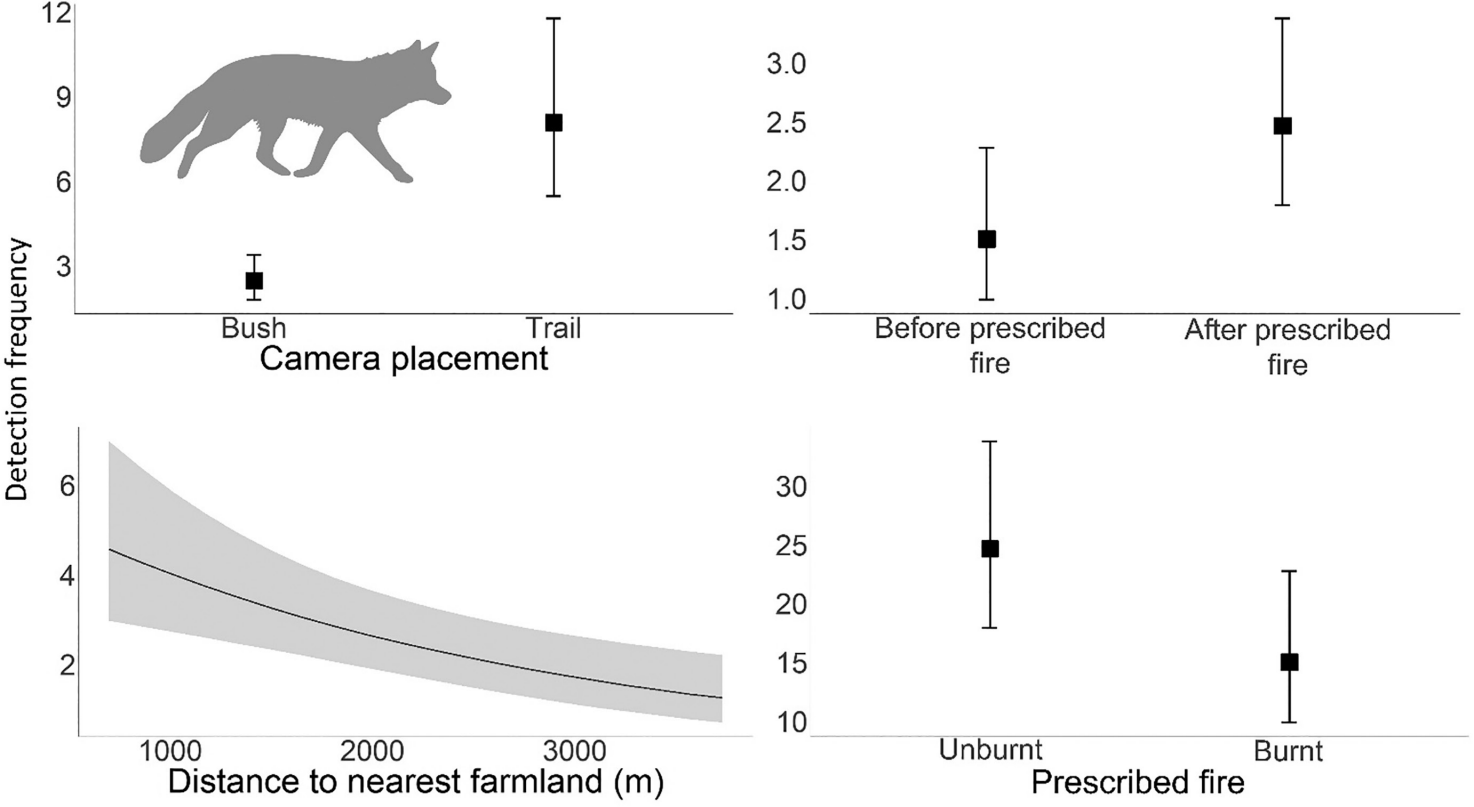
Plots of the GLMMs showing the influential effects from the two well‐supported models (ΔAIC < 2) on the activity (detection frequency) of the European red fox (*Vulpes vulpes*) in the eastern Otway Ranges, Victoria. Full model summaries are provided in Appendix S3.

There were eight well‐supported models for foxes (Appendix S3). Fox activity was higher on trails compared to off trails (1.24 [0.82, 1.67]; 8/8 models), higher after the prescribed fire across all sites (−0.50 [−0.90, −0.10]; 8/8 models), higher at unburnt compared to burnt sites (0.57 [0.13, 1.02]; 8/8 models), and negatively associated with distance from farmland (−0.35 [−0.55, −0.15]; 5/8 models) (Figure 3). There was no evidence that fox activity was influenced by prey activity (Appendix S3).

### 3.3 Predictors of macropod activity

**FIGURE 4:**
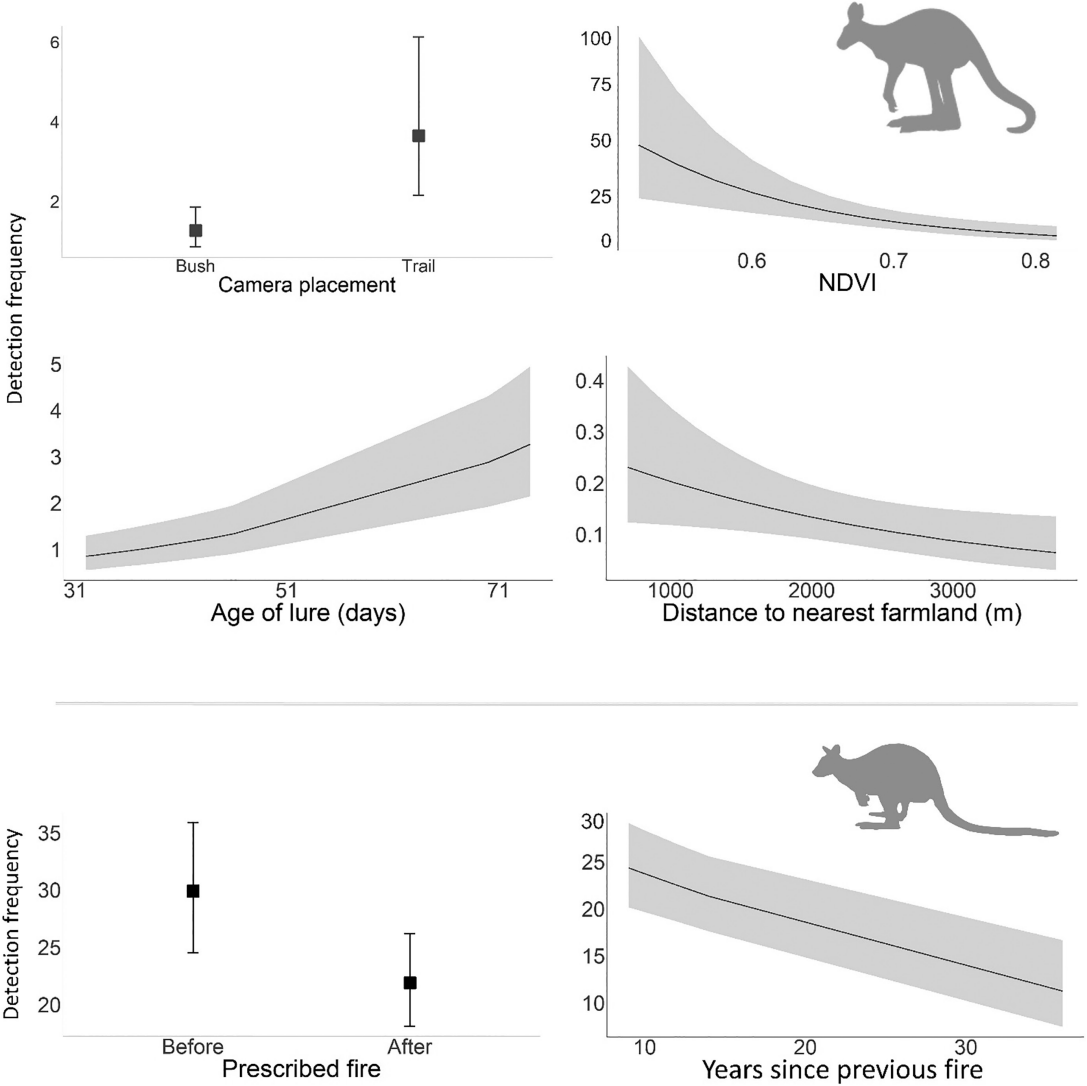
Influential effects on activity (detection frequency) from the single, well‐supported models (ΔAIC < 2) for both the eastern grey kangaroo (*Macropus giganteus*) (top four plots) and the swamp wallaby (*Wallabia bicolor*) (bottom two plots) (Appendix S3).

There were six well‐supported models for the eastern grey kangaroo (Appendix S3). Kangaroo activity was higher on trails (1.09 [0.43, 1.74]; 6/6 models), positively associated with lure age (0.48 [0.34, 0.61]; 6/6 models), and negatively associated with both NDVI (−0.45 [−0.70, −0.21]; 6/6 models) and distance from farmland (−0.34 [−0.64, −0.04]; 5/6 models) (Figure 4). There was no evidence that kangaroo activity was influenced by the prescribed fire (Appendix S3). There were two well‐supported models for the swamp wallaby, both of which indicated that activity declined with Years since previous fire (−0.32 [−0.47, −0.16]) and was higher before the prescribed fire across all sites (0.42 [0.10, 0.74]) (Figure 4).

### 3.4 Predictors of small‐ and medium‐sized mammal activity

**FIGURE 5:**
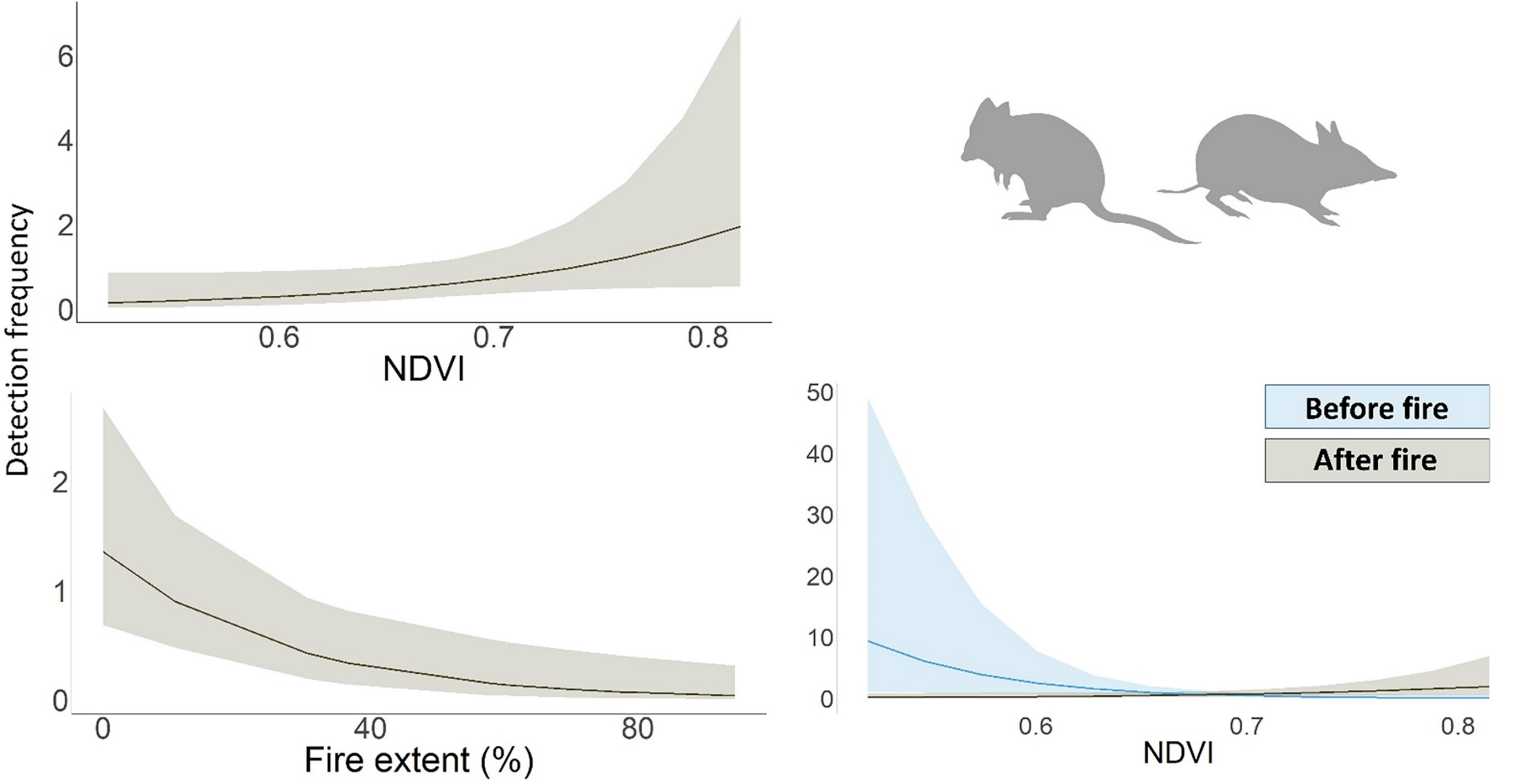
Plots of the GLMMs showing the influential effects from the single, well‐supported models (ΔAIC < 2) on the activity (detection frequency) of medium‐sized mammals in the eastern Otway Ranges, Victoria. Full model summaries are provided in Appendix S3.

There were four well‐supported models for the medium‐sized mammal group (Appendix S3). Activity was negatively associated with Fire extent (−1.17, [−1.92, −0.42]; 4/4 models) and NDVI before the prescribed fire (−1.42 [−2.11, −0.74]; 4/4 models) (Figure 5). Activity was also positively associated with the main effect of NDVI (0.55 [0.03, 1.06]; 2/4 models) (Figure 5). There were nine well‐supported models for the small mammal group, however, none of these contained influential effects of the predictor variables (i.e., the confidence intervals of all predictor variables overlapped zero). The null model was the second highest‐ranked model (ΔAIC = 0.32).

## 4 DISCUSSION

We examined the effect of prescribed fire and its interaction with key environmental variables on the activity of the invasive feral cat and red fox, and the native mammal community within a high‐value conservation reserve in south‐eastern Australia. Neither foxes nor cats were strongly influenced by the prescribed fire or prey availability, although fox activity was notably higher near farmland and cats were more active in sites with fewer years since previous fire, partially supporting Prediction 1. Similarly, swamp wallaby activity was higher in sites with fewer years since previous fire, but their activity decreased across all sites following the prescribed burn, providing mixed support for Prediction 2. Medium‐sized mammal activity was positively associated with areas of high productivity and negatively associated with prescribed fire extent, lending some support to Prediction 3. These results provide further evidence of the complex and likely context‐dependent relationships between cats, foxes, native mammals, and fire.

### 4.1 Cats and foxes

We predicted that cat and fox activity would be higher in areas burnt by the prescribed fire, however, we found no strong evidence to support this. Although some studies have found strong evidence of cat or fox activity increasing after fire (Birtsas et al., 2012; McGregor, Cliff, & Kanowski, 2016; Miritis et al., 2023), others have found a negative response (Alexandre et al., 2020; Bird et al., 2018; Lothian et al., 2022), no response (Hradsky, Robley, et al., 2017; Moore et al., 2018; Senior et al., 2022), or a context‐dependent response. For instance, in the USA, swift foxes (*V. velox*), which are closely related to red foxes, only used burnt areas more frequently if their existing core home ranges were burnt (Thompson et al., 2008). Indeed, a recent analysis of existing evidence from Australia found that there was a high likelihood of neutral responses to fire being recorded for both cats and foxes in Australia (55% and 67%, respectively; Doherty et al., 2023).

One explanation for our result could be that the prescribed fire in this study was too small and/or mild to elicit an increase in cat or fox activity (e.g., Pons & Bas, 2005; Puig‐Gironès et al., 2022). Notably, however, Doherty et al. (2023) found that fire type (wildfire vs. prescribed fire) did not influence cat responses, whereas fox activity was more likely to increase after prescribed burns and decrease after wildfires. This indicates that the response of these species to fire is not determined solely by fire type or severity, but rather a complex interplay of factors that likely include pre‐fire environmental conditions, prey availability, and ecosystem‐specific dynamics. For instance, cat abundance declined considerably following a severe wildfire in southern Australia (Hohnen et al., 2021). In contrast, a GPS study in northern Australia found that cats strongly selected for areas recently burnt by severe prescribed fires and containing high prey abundance (McGregor et al., 2014). However, they did not select for areas recently burnt by mild fire, despite these areas having a high abundance of small mammal prey (McGregor et al., 2014). The authors suggested that cats did not benefit from mildly burnt areas due to unburnt patches likely providing refuge for prey, making hunting less profitable compared to severely burnt areas (McGregor et al., 2014).

Although cats did not increase their activity in the short‐term following the prescribed fire, they were more active in areas with fewer years since the previous fire and lower NDVI before the fire—both indicators of simpler vegetation structure (Haslem et al., 2016). Cats also favoured areas with a higher NDVI after the fire and swampy riparian woodlands, which is typically the most structurally complex vegetation type in our study area. These seemingly conflicting habitat preferences may be explained by resource availability. Feral cats are capable of exploiting a diverse range of habitats, and it is generally considered that they prefer dense habitats for shelter—such as riparian woodlands—and more open habitats for hunting (Doherty, Bengsen, & Davis, 2015; Lozano et al., 2003; Stobo‐Wilson, Stokeld, et al., 2020). Although foraging efficiency may theoretically be highest in recently burnt areas (McGregor et al., 2015), these areas may be sub‐optimal, and thus avoided, if prey availability is low (Pyke et al., 1977). The small mammal population in the eastern Otway Ranges has been dramatically declining for several decades (Wayne et al., 2017; Wilson & Aberton, 2006; Wilson et al., 2001, 2017; Wilson & Garkaklis, 2020). Although we did not find a relationship between fire and small mammal activity (discussed below under *Small‐ and medium‐sized mammals*), other studies have found that our most frequently detected small mammal species, the bush rat, almost completely avoids recently burnt areas (Lees et al., 2022). Further, during a separate, concurrent small mammal study in the eastern Otways, we found that detections of other small mammal species in burnt areas were very uncommon (Watchorn, Doherty, et al., in prep). These factors may partially explain why neither cats nor foxes increased their activity in burnt areas. It may also explain why cat activity was higher in relatively complex areas, as they likely supported higher prey availability (Lees et al., 2022; Swan et al., 2016).

We did, however, find that fox activity increased across the study area after the prescribed fire. Although this broadly aligns with observations from the foxes' native range in the Mediterranean (e.g., Birtsas et al., 2012; Puig‐Gironès & Pons, 2020), due to the proximity of our control and treatment sites in our study we cannot be sure if the prescribed fire drove this change. Juvenile foxes typically disperse in winter (April to June in this region; Baker et al., 2001), and Hradsky, Penman, et al. (2017) also observed an increase in fox activity at their control sites following a prescribed fire, which was thought to be driven by dispersing foxes. Spatially independent control sites would have better isolated the impact of the fire from other factors like seasonal fox dispersal if the sampling design was able to avoid spatial confounding at such a large scale, however, equipment limitations made such an approach unfeasible for this study.

We predicted that cat and fox activity would be higher near farms and towns due to increased resource availability (Doherty, Bengsen, & Davis, 2015; Ferreira et al., 2011; Hradsky, Robley, et al., 2017). We found some support for this prediction, with foxes more active closer to farmland. Across their global range, red foxes, as well as other generalist predators such as coyotes (*Canis latrans*) and golden jackals (*C. aureus*), use farmlands due to the availability of denning habitat, water and food (e.g., livestock, rodents, human food waste), as well as edge habitats which can support relatively diverse fauna communities and hunting opportunities (Aikawa & Saito, 2023; Gosselink et al., 2003; Laux et al., 2022; Šálek et al., 2014). Indeed, we found that the eastern grey kangaroo, a common prey item of the red fox (Stobo‐Wilson et al., 2022), was also more active near farmlands—likely due to the availability of pasture for grazing (Arnold et al., 1992; Maguire et al., 2006). In a GPS tracking study, Hradsky, Robley, et al. (2017) found that foxes in the Otway Ranges selected for farmland and forest–farmland interfaces at night, possibly due to livestock carcasses or prey availability. Collectively, these findings indicate that farm peripheries could provide effective fox‐baiting targets for land managers (Carter & Luck, 2013; Engeman & Linnell, 1998). Further high‐resolution telemetry and resource mapping studies may provide further insight into the specific features foxes use (e.g., water dams, livestock carcasses), which may further improve bait uptake in these areas.

### 4.2 Macropods

Fire stimulates a short‐term increase in plant nutrients, such as nitrogen (N), increasing both the nutritional availability and palatability for herbivores (Eby et al., 2014). This, in turn, can drive temporary increases in the abundance of large herbivores after fire—a phenomenon observed around the world (Klop et al., 2007; Raynor et al., 2015; Reid et al., 2023). However, in environments with large cursorial predators, such as coyotes, foxes, and wolves, herbivores must also contend with the increased risk of exposure, and thus predation, in these recently burnt areas (Cherry et al., 2017; Doherty et al., 2022; Joly et al., 2003). In our study, swamp wallaby activity decreased across all sites following the prescribed burn, partially supporting our second prediction. Indeed, the activity of foxes—which are known to prey on swamp wallabies (Wolrige, 2000)—increased across our study area after the prescribed fire. These results are in line with those of Hradsky, Penman, et al. (2017), who found that foxes decreased their consumption of swamp wallabies after prescribed fire but increased their consumption of smaller mammals; possibly because the wallabies were considerably more mobile and had dispersed away from the burnt area to reduce predation risk. Di Stefano et al. (2009) also found that swamp wallabies favour more structurally complex vegetation to increase obscurity from predators. Similarly, Banks (2001) found that eastern grey kangaroos were more active in open areas where foxes had been removed and spent more time near forest edges when foxes were present, although we did not detect a response of kangaroo to fire in our study.

We did, however, find that swamp wallaby activity was higher in areas with fewer years since fire, a result consistent with other research showing this species' preference for early successional vegetation (Chard et al., 2022; Styger et al., 2011). This initially seems contradictory to our observed decline of wallaby activity after the prescribed fire. However, the reduction in canopy cover caused by fire can reinvigorate the understory and facilitate the growth of grasses and forbs—preferred by macropods for browsing—in the years following (Whelan, 1995). After the initial period of vegetation recovery and heightened exposure to predators (e.g., 0–12 months, although this is dependent on fire severity, rainfall, etc.), the regenerating burnt area may provide suitable grazing habitat for wallabies whilst also providing enough lateral vegetation cover to reduce the perceived predation risk (Di Stefano et al., 2009; Di Stefano & Newell, 2008; Leonard et al., 2010; Swan et al., 2009).

### 4.3 Small‐ and medium‐sized mammals

The small‐ and medium‐sized mammals in our study area typically prefer dense vegetation, where food and denning resources are greater and the risk of predation is likely lower (Catling et al., 2001; Dexter et al., 2011). We therefore predicted that the activity of these groups will be positively associated with NDVI and decrease after the prescribed fire (White et al., 2022). In partial support of this prediction, the activity of medium‐sized mammals decreased with Fire extent. This negative response has been observed in other mammals of similar size and ecological requirements in Europe (e.g., Sokos et al., 2016), North America (e.g., Zwolak & Foresman, 2007), and elsewhere in Australia (e.g., Robley et al., 2023). Hradsky, Mildwaters, et al. (2017) observed that fox consumption of medium‐sized mammals that typically prefer densely vegetated areas, such as bandicoots, doubled after a patchy prescribed fire in the Otways, highlighting the increased vulnerability of these species to predation following fire.

Medium‐sized mammals also showed a complex relationship with NDVI. Activity was negatively associated with NDVI before the fire and slightly increased afterwards, suggesting that these species increased their selection of microhabitat such as gullies and sedges following the fire (e.g., Fordyce et al., 2016; Lees et al., 2022; Swan et al., 2016), although there considerable uncertainty with this prediction. As with the fox, however, this change was observed across both burnt and unburnt sites. It is possible that the increased selection for NDVI post‐fire was a temporal response to the increase in juvenile fox activity, although spatially independent control sites would have allowed us to better isolate the impact of the fire. We also observed the same relationship between cat activity and NDVI before and after fire. This elevated prey activity in these areas may explain why cat activity was higher in relatively complex areas after the prescribed fire.

Notably, although higher NDVI is typically associated with higher species richness for the mammals comprising our medium‐sized mammal group (Dorph et al., 2021; White et al., 2022; Youngentob et al., 2015), individually, these species have exhibited positive (Miritis et al., 2020; White et al., 2022), negative (Ralph, 2021), and neutral (Hale et al., 2016; Youngentob et al., 2015) responses to NDVI. These varied relationships are likely due to factors such as drought, fire history, and habitat type (White et al., 2022; Youngentob et al., 2015). Nonetheless, our findings suggest that NDVI may be a useful means of identifying and conserving productive and structurally complex areas which may facilitate mammal occurrence and diversity in this landscape (Rivarola, 2022; Sukma et al., 2019), especially following disturbances such as drought (White et al., 2022), plant pathogen spread (Casey, 2022), or fire (Dorph et al., 2021).

None of the variables predicted the activity of small mammals, a finding at odds with previous studies on these species that identified relationships with NDVI (Chadwick et al., 2022; Hale et al., 2016) and changes in habitat use following fire (Fordyce et al., 2016; Lees et al., 2022; Swan et al., 2016). One reason for this discrepancy may be that our camera arrangement, in terms of both density and spatial extent, was insufficient to reliably detect discrete patterns of microhabitat use for these species, especially considering the depauperate state of the small mammal community (Wilson & Garkaklis, 2020). The incorporation of fine‐scale, site‐level habitat information—such as understorey vegetation structure (e.g., Hradsky, Mildwaters, et al., 2017; Lees et al., 2022)—may have further improved our ability to detect any potential response to fire or vegetation structure.

### 4.4 Management implications

The lack of a strong response from cats and foxes to the prescribed fire in this study challenges the generalisation that fire elevates their impact in burnt areas. When contextualising our results within the broader literature, it appears that their response is variable and likely dependent on factors such as the scale and severity of the fire, as well as prey availability. It is also likely sensitive to study design (see Section 4.5). This emphasises the importance of drawing on a breadth of information when developing management plans. Despite the lack of a clear relationship with fire, we nonetheless recommend the intensive lethal management of these species after fire (e.g., Johnston, 2012), given the considerable risk they pose to native fauna (Stobo‐Wilson et al., 2021, 2022; Woinarski et al., 2015) and the potential for an immensely damaging outcome should there be a synergistic interaction with fire (Doherty, Dickman, et al., 2015). Indeed, it is possible that the decline in medium‐sized mammal activity in extensively burnt areas was attributable to cats and foxes (e.g., Hradsky, Mildwaters, et al., 2017) and our study design lacked the sensitivity to detect this.

Additionally, the negative effect of the prescribed fire on native mammals, along with the positive association of medium‐sized mammals with productive vegetation, highlights the importance of retaining unburnt, structurally complex, high NDVI areas within the landscape (Kelly et al., 2012). This will become increasingly important as the reliance on prescribed burns for reducing wildfire risk continues to grow and the proportion of long‐unburnt habitats continues to diminish (Kolden, 2019; Spies et al., 2006; Varner et al., 2021).

Moreover, the use of lures with camera traps can influence animal behaviour, which may complicate the interpretation of their response to fire (Gil‐Sánchez et al., 2021; Johnson et al., 2021). For instance, da Rocha et al. (2016) found that lures had no effect on carnivore capture rates in Brazil, but likely reduced prey species capture rates. It is possible that the use of lures may have skewed the activity patterns of certain species resulting in higher or lower frequency of detection in our study, and this should be considered when interpreting our results.

### 4.5 Future research questions

There are several key research avenues which should be prioritised to improve the conservation of fauna in protected areas after fire. The majority of evidence demonstrating increases in cat activity post‐fire comes from Australia's tropical north (Doherty et al., 2023). We have little clear evidence to answer the question: do cats and foxes temporarily adjust their core home range or move long distances to hunt in recently burnt areas in the temperate forests and woodlands of southern Australia?

Although camera traps are indeed useful, their appropriateness for answering this question is limited by their inherently patchy spatial coverage across the landscape, as well as the delay associated with deploying them immediately after prescribed fires, especially in temperate forests. To this end, we recommend future studies employ the BACI experimental framework with animal‐borne GPS loggers (Le Pla et al., 2023). This will improve our understanding of the fine‐scale movements and habitat use of cats, foxes, and native prey species immediately before and after prescribed fire (e.g., McGregor et al., 2014). Animal‐born video cameras could also provide valuable insight into cat and fox hunting success and prey selection (e.g., McGregor et al., 2015). These approaches, although challenging to implement, will allow for a more nuanced understanding of fine‐scale responses to prescribed fire, thereby informing more effective management strategies within protected areas.

We also encourage the continued investigation into the efficacy of providing artificial refuges (Watchorn et al., 2022) for small native fauna in recently burnt areas (Agnew, 2022; Bleicher & Dickman, 2020; Hegarty, 2022; Watchorn, Dickman, et al., 2024; Watchorn, Doherty, et al., in prep). Although still in its infancy, this approach may help to improve the persistence of small fauna after fire—in the presence of cats and foxes—by providing supplementary refuge habitat which excludes these predators. However, further research is still required to determine whether this can improve fauna survival or abundance after fire.

## AUTHOR CONTRIBUTIONS

**Darcy J. Watchorn:** Conceptualization (equal); data curation (lead); formal analysis (lead); investigation (lead); methodology (lead); project administration (equal); validation (lead); visualization (lead); writing – original draft (lead); writing – review and editing (lead). **Tim S. Doherty:** Conceptualization (equal); data curation (supporting); formal analysis (supporting); funding acquisition (lead); investigation (supporting); methodology (supporting); project administration (supporting); resources (lead); supervision (supporting); validation (supporting); visualization (supporting); writing – original draft (supporting); writing – review and editing (supporting). **Barbara A. Wilson:** Conceptualization (supporting); funding acquisition (supporting); investigation (supporting); methodology (supporting); project administration (supporting); resources (supporting); supervision (supporting); validation (supporting); visualization (supporting); writing – original draft (supporting); writing – review and editing (supporting). **Mark J. Garkaklis:** Conceptualization (supporting); funding acquisition (supporting); investigation (supporting); methodology (supporting); project administration (supporting); resources (supporting); supervision (supporting); validation (supporting); visualization (supporting); writing – original draft (supporting); writing – review and editing (supporting). **Don A. Driscoll:** Conceptualization (supporting); data curation (supporting); formal analysis (supporting); funding acquisition (supporting); investigation (supporting); methodology (supporting); project administration (supporting); resources (supporting); supervision (lead); validation (supporting); visualization (supporting); writing – original draft (supporting); writing – review and editing (supporting).

## FUNDING INFORMATION

This work was supported by funding from the Hermon Slade Foundation (HSF 18/5). Open access publishing facilitated by Deakin University, as part of the Wiley ‐ Deakin University agreement via the Council of Australian University Librarians.

## CONFLICT OF INTEREST STATEMENT

The authors confirm that there are no conflicts of interest.

## Supporting information


Appendix S1.

Appendix S2.

Appendix S3.

## Data Availability

The data that supports the findings of this study is permanently archived in OSF (https://osf.io/vgae8/?view_only=8f3be5d46dd0414186dce2616cc466c6).
