# Supplementary material for: How do invasive predators and their native prey respond to prescribed fire?
Source: Ecol Evol. 2024 May 22;14(5):e11450. doi: 10.1002/ece3.11450 (PMC11112300; doi:10.1002/ece3.11450)
Supplement: Supplementary file 1 — Appendix S1. [file ECE3-14-e11450-s002.docx]

# Appendix S1: Additional survey details

**Appendix S1: Table S1.** The timing and duration of each survey period in relation to the prescribed fire in the north-eastern Otway Ranges, Victoria, which was ignited in mid-May 2019. Survey length ranges are shown in parentheses, along with the approximate season of the survey period.

| Survey | | Mean survey length (range) | Time of year  (season) |
| --- | --- | --- | --- |
| 1 | 6-months pre-fire | 71 days (55 – 74 days) | October – December 2018  (Late spring–early summer) |
| 2 | 2-months pre-fire | 64 days (54 – 65 days) | February – April 2019  (Late summer–early autumn) |
| 3 | 2-weeks post-fire | 64 days (61 – 66 days) | May – July 2019  (Late autumn–mid-winter) |
| 4 | 3-months post-fire | 64 days (64 – 64 days) | July – October 2019  (Mid-winter–mid-spring) |
| 5 | 6-months post-fire | 64 days (63 – 64 days) | October – December 2019  (Mid-spring–early summer) |

**Appendix S1: Table S2.** The number of individual mammal detections per survey during the study period. The koala, red-necked wallaby, and short-beaked echidna were not included in the analysis due to their small number of detections.

| Group | Species | 6-months pre-fire | 2-months pre-fire | 2-weeks post-fire | 3-months post-fire | 6-months post-fire | Total |
| --- | --- | --- | --- | --- | --- | --- | --- |
| Invasive predators | Red fox,  *Vulpes vulpes* | 59 | 42 | 71 | 54 | 60 | **286** |
|  | Feral cat,  *Felis catus* | 49 | 19 | 25 | 19 | 20 | **132** |
| Macropods | Eastern grey kangaroo,  *Macropus giganteus* | 49 | 39 | 21 | 53 | 128 | **290** |
|  | Swamp wallaby,  *Wallabia bicolor* | 982 | 675 | 461 | 450 | 663 | **3231** |
| Medium mammals (800 - 2,000 g) | Common brushtail possum, *Trichosurus vulpecula* | 3 | 9 | 7 | 11 | 19 | **49** |
|  | Eastern ringtail possum, Pseudocheirus peregrinus | 7 | 8 | 7 | 12 | 13 | **47** |
|  | Long-nosed bandicoot, *Perameles nasuta* | 11 | 12 | 3 | 7 | 1 | **34** |
|  | Long-nosed potoroo,  *Potorous tridactylus* | 8 | 7 | 23 | 11 | 8 | **57** |
|  | Southern brown bandicoot,  *Isoodon obesulus* | 0 | 1 | 4 | 1 | 0 | **6** |
| Small mammals  (<800 g) | Agile antechinus,  *Antechinus agilis* | 7 | 15 | 23 | 8 | 2 | **55** |
|  | Bush rat,  *Rattus fuscipes* | 14 | 20 | 2 | 48 | 9 | **93** |
|  | Eastern pygmy possum,  *Cercartetus nanus* | 2 | 3 | 0 | 0 | 0 | **5** |
|  | Krefft's glider,  *Petaurus notatus* | 0 | 1 | 1 | 1 | 0 | **3** |
|  | Swamp antechinus,  *Antechinus minimus maritimus* | 0 | 2 | 1 | 0 | 0 | **3** |
|  | White-footed dunnart,  *Sminthopsis leucopus* | 0 | 3 | 0 | 0 | 0 | **3** |
|  | Unknown small mammal species | 0 | 0 | 1 | 9 | 0 | **10** |
