# Supplementary material for: How do invasive predators and their native prey respond to prescribed fire?
Source: Ecol Evol. 2024 May 22;14(5):e11450. doi: 10.1002/ece3.11450 (PMC11112300; doi:10.1002/ece3.11450)
Supplement: Supplementary file 2 — Appendix S2. [file ECE3-14-e11450-s003.docx]

**Appendix S2: Single-species occupancy models**

Given the challenges in accommodating potentially elevated detection probabilities (*p*) in burnt areas within our GLMMs, we opted for fitting a single-species occupancy model to each of our six species/groups to test the effect of Treatment on detectability. We did this using the ‘occu’ function from the unmarked package (Kellner et al. 2023; Fiske and Chandler 2011) in R version 4.2.2 (R Core Team 2022). Due to the modest number of detections and associated model overfitting issues, we were unable to test the interaction of Treatment (Burnt *cf.* Unburnt sites) and Time (Before *cf.* After fire) in a single model. To overcome this issue whilst still accounting for the effect of Time, we fitted a hybrid Treatment variable consisting of four levels: Burnt (before fire), Unburnt (before fire), Burnt (after fire), and Unburnt (after fire). There were many days when each species/group were not detected at a given site, meaning that the dataset had a high number of zeros. To accommodate this and to avoid overfitting the models, we aggregated survey data into 10-day periods, assigning a ‘1’ for detection within a 10-day period and a ‘0’ otherwise. We assumed occupancy (ψ) to be constant in these models, as our primary objective was to assess how Treatment influenced detection probability, rather than occupancy *per se*.

We found that both the swamp wallaby and small mammal group had a higher probability of detection before the fire (Figure S1, S2; Table S1). However, detection probably was not higher in burnt areas for any of the six species groups (Table S1). We, therefore, used GLMMs to test the effect of our predictor variables (Table 1) on mammal activity, as they provide a more flexible approach better suited to addressing our research questions, whilst also allowing us to incorporate more data.

**
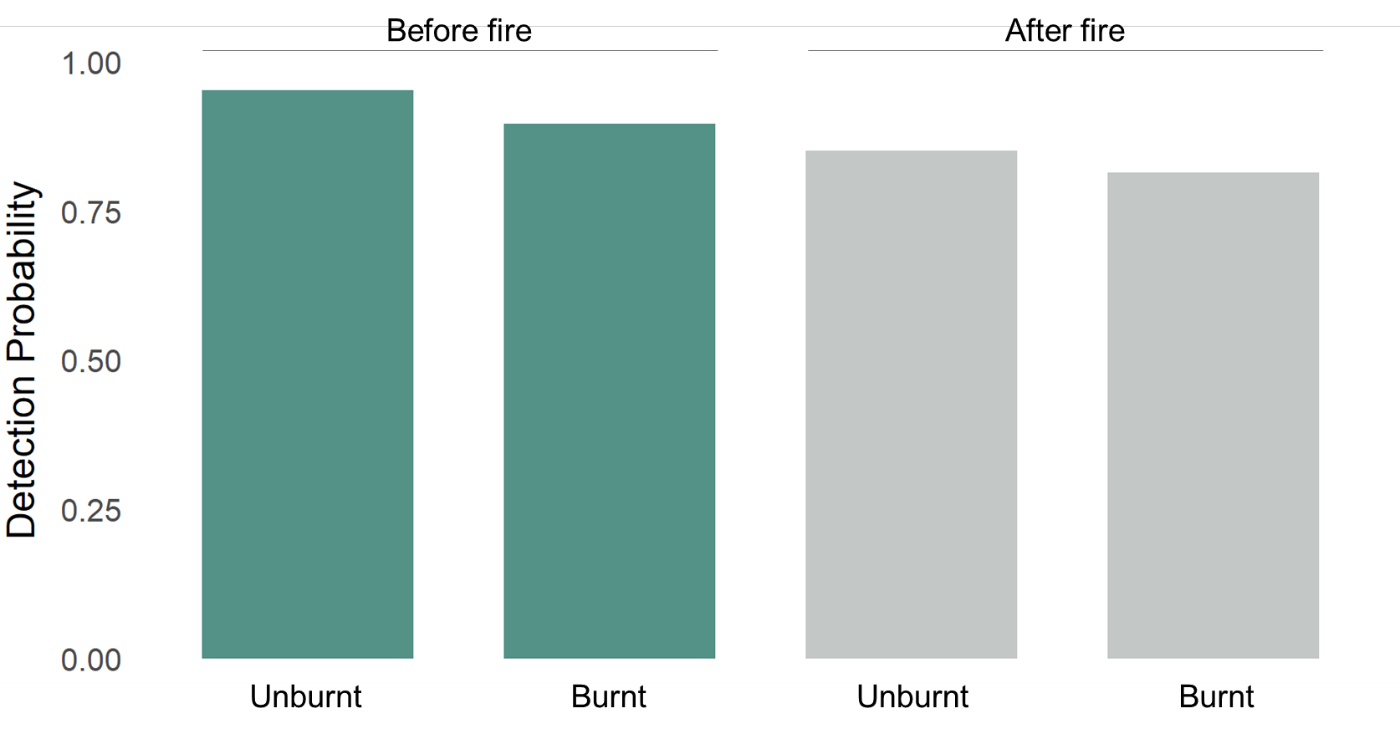
**

**Figure S1.** Estimates of detection probably for the swamp wallaby (*Wallabia bicolor*) before and after the 2019 prescribed fire in the Otway Ranges, at both burnt and unburnt sites.


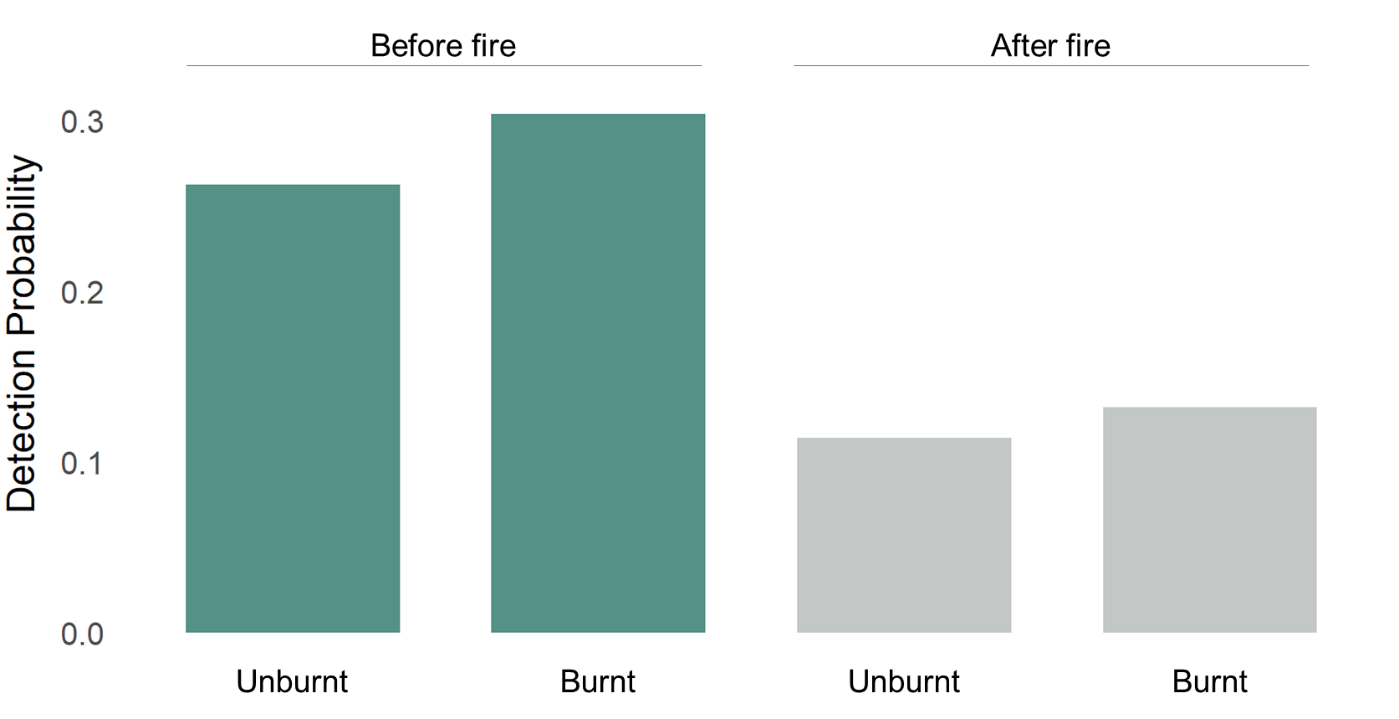


**Figure S2.** Estimates of detection probably for the small mammal species group before and after the 2019 prescribed fire in the Otway Ranges, at both burnt and unburnt sites

**Table S1.** Model summaries of the occupancy models fitted to test for the effect of the prescribed fire on detection probability. Model terms with strong evidence for an effect on detectability are shown in **bold text**.

| Model | Term | Estimate | SE | z | P-value |
| --- | --- | --- | --- | --- | --- |
| Red fox | Intercept | -1.08 | 0.23 | -4.69 | 0.00 |
|  | Burnt (Before fire) | 0.57 | 0.30 | 1.89 | 0.06 |
|  | Unburnt (After fire) | 0.06 | 0.27 | 0.22 | 0.83 |
|  | Unburnt (Before fire) | 0.16 | 0.39 | 0.40 | 0.69 |
| Feral cat | Intercept | -1.28 | 0.36 | -3.56 | 0.00 |
|  | Burnt (Before fire) | -0.07 | 0.51 | -0.14 | 0.89 |
|  | Unburnt (After fire) | 0.26 | 0.40 | 0.65 | 0.52 |
|  | Unburnt (Before fire) | 0.00 | 0.48 | 0.00 | 1.00 |
| Swamp wallaby | Intercept | 1.48 | 0.18 | 8.46 | 0.00 |
|  | **Burnt (Before fire)** | **0.67** | **0.32** | **2.07** | **0.04** |
|  | Unburnt (After fire) | 0.26 | 0.23 | 1.12 | 0.26 |
|  | **Unburnt (Before fire)** | **1.50** | **0.36** | **4.22** | **<0.01** |
| Eastern grey kangaroo | Intercept | -1.33 | 0.27 | -5.01 | 0.00 |
|  | Burnt (Before fire) | 0.41 | 0.39 | 1.05 | 0.29 |
|  | Unburnt (After fire) | 0.78 | 0.31 | 2.55 | 0.01 |
|  | Unburnt (Before fire) | 0.77 | 0.33 | 2.35 | 0.02 |
| Small mammals | Intercept | -1.88 | 0.34 | -5.60 | 0.00 |
|  | **Burnt (Before fire)** | **1.05** | **0.43** | **2.47** | **0.01** |
|  | Unburnt (After fire) | -0.17 | 0.45 | -0.37 | 0.71 |
|  | **Unburnt (Before fire)** | **0.85** | **0.41** | **2.05** | **0.04** |
| Medium mammals | Intercept | -0.23 | 0.44 | -0.51 | 0.61 |
|  | Burnt (Before fire) | -0.06 | 0.67 | -0.08 | 0.93 |
|  | Unburnt (After fire) | 0.17 | 0.62 | 0.28 | 0.78 |
|  | Unburnt (Before fire) | 0.16 | 0.53 | 0.31 | 0.76 |

**References**

Fiske, Ian, and Richard Chandler. 2011. “Unmarked: An R Package for Fitting Hierarchical Models of Wildlife Occurrence and Abundance.” *Journal of Statistical Software* 43: 1–23.

Kellner, Kenneth F, Adam D Smith, J Andrew Royle, Marc Kéry, Jerrold L Belant, and Richard B Chandler. 2023. “The Unmarked R Package: Twelve Years of Advances in Occurrence and Abundance Modelling in Ecology.” *Methods in Ecology and Evolution*.
